# Role of Intestinal Ultrasound in the Management of Patients with Inflammatory Bowel Disease

**DOI:** 10.3390/life11070603

**Published:** 2021-06-23

**Authors:** Aranzazu Jauregui-Amezaga, Jordi Rimola

**Affiliations:** 1 Service of Gastroenterology, University Hospital Antwerp—University of Antwerp, 2650 Edegem, Belgium; 2 IBD Unit, Service of Radiology, Clinic Hospital Barcelona, 08036 Barcelona, Spain; jrimola@clinic.cat

**Keywords:** IUS, bowel ultrasound, inflammatory bowel diseases, IBD, Crohn’s disease, ulcerative colitis, imaging, cross-sectional imaging

## Abstract

Intestinal ultrasound (IUS) has gained popularity as a first line technique for the diagnosis and monitoring of patients with inflammatory bowel diseases (IBD) due to its many advantages. It is a non-invasive imaging technique with non-ionizing radiation exposure. It can be easily performed not only by radiologists but also by trained gastroenterologists at outpatient clinics. In addition, the cost of IUS equipment is low when compared with other imaging techniques. IUS is an accurate technique to detect inflammatory lesions and complications in the bowel in patients with suspected or already known Crohn’s disease (CD). Recent evidence indicates that IUS is a convenient and accurate technique to assess extension and activity in the colon in patients with ulcerative colitis (UC), and can be a non-invasive alternative to endoscopy. In patients with IBD, several non-specific pathological ultrasonographic signs can be identified: bowel wall thickening, alteration of the bowel wall echo-pattern, loss of bowel stratification, increased vascularization, decreased bowel peristalsis, fibro-fatty proliferation, enlarged lymph nodes, and/or abdominal free fluid. Considering the transmural CD inflammation, CD complications such as presence of strictures, fistulae, or abscesses can be detected. In patients with UC, where inflammation is limited to mucosa, luminal inflammatory ultrasonographic changes are similar to those of CD. As the technique is related to the operator’s experience, adequate IUS training, performance in daily practice, and a generalized use of standardized parameters will help to increase its reproducibility.

## 1. Introduction

Although colonoscopy is the modality of choice to assess disease activity of patients with inflammatory bowel diseases (IBD), intestinal ultrasound (IUS) may be used as an alternative to evaluate disease activity, providing relevant additional information related to the extension and presence of complications in patients with Crohn’s disease (CD) [1]. This information is key to guide therapy decisions.

## 2. Standard Intestinal Ultrasound

Intestinal ultrasound is a non-invasive imaging technique that is widely available and does not use ionizing radiation. It is easy to use and well tolerated by patients. It allows the direct visualization of most colonic segments and ileum, and the assessment of bowel wall layers, motility, and vascularization [1].

**Figure 1:**
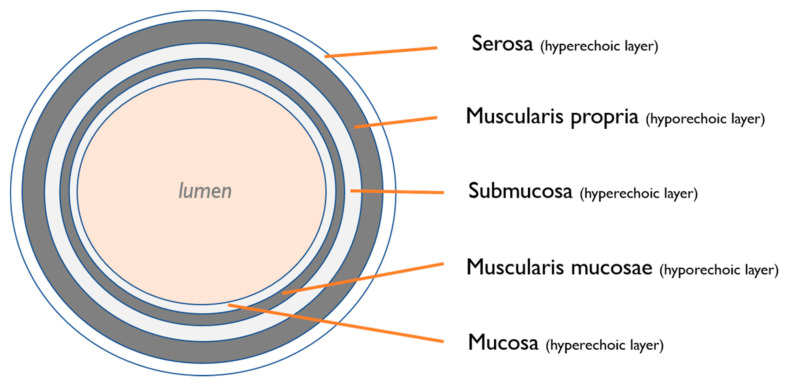
Bowel wall layers: The bowel wall layers are, from the bowel lumen: (a) the mucosal layer (hyperechoic), which is the interface between the mucosa and the bowel lumen; (b) the deep mucosa (hypoechoic), which has a variable thickness and represents the packed glandular tissue; (c) the submucosa (hyperechoic), which contains connective tissue with vessels, nerves and fat; (d) the muscularis propria (hypoechoic), with an inner circular muscle layer and an outer longitudinal muscle layer; and (e) the serosa (hyperechoic), which is the visceral peritoneum.

The bowel wall layers are, from the bowel lumen: [2] (a) the mucosal layer (hyperechoic), which is the interface between the mucosa and the bowel lumen; (b) the deep mucosa (hypoechoic), which has a variable thickness and represents the packed glandular tissue; (c) the submucosa (hyperechoic), which contains connective tissue with vessels, nerves, and fat; (d) the muscularis propria (hypoechoic), with an inner circular muscle layer and an outer longitudinal muscle layer; and (e) the serosa (hyperechoic), which is the visceral peritoneum (see Figure 1).

There is no need for fasting, bowel preparation, or oral/intravenous contrast for a standard IUS examination using the B-mode. Fasting for 4–6 h decreases bowel motility while oral water of oral contrast with a polyethylene glycol solution can be used to improve peristalsis and visualization of the small bowel [3]. The patient should be in a supine position. It is recommended to perform an initial systematic scan (panoramic view of the entire abdomen) with a convex probe (low frequency 5–8 MHz) and continue with a detailed visualization of the intestinal wall (the 5-layer pattern) using a linear probe (high frequency 11–14 MHz) [4,5]. Color Doppler US can assess the signal from the small vessels at the intestinal wall and it should be used to assess vascularization of pathological segments [4].

## 3. Advanced Intestinal Ultrasound Techniques

Other types of IUS examinations may increase the accuracy of the technique, such as small intestine contrast ultrasonography, contrast- enhanced ultrasonography, and elastography. On the other hand, they also increase the duration and invasiveness of the technique in clinical practice, so they should not be performed regularly in daily clinical practice.

Small intestine contrast ultrasonography (SICUS) is performed after the administration of an oral contrast agent such as polyethylene glycol (PEG) solution (500–800 mL) approximately 30–40 min before the examination. It is time-consuming since the oral contrast has to be visualized in the caecum in order to perform correct retrograde examination, but there is an improvement in the visualization of the small bowel loops filled with oral contrast that increases the localization, extension, and detection of stricturing and penetrating complications [6,7,8,9,10].

Contrast-enhanced ultrasonography (CEUS) requires the administration of intravenous contrast agents (bolus injection of stabilized microbubbles with gaseous content) into the blood stream that allows real-time visualization of the small bowel microvasculature [4]. The only accepted use of CEUS is the differentiation between abscess and inflammatory masses and also facilitates the delimitation of fluid cavities that would facilitate an eventual percutaneous drainage [1]. However, some authors have explored additional uses of CEUS, including characterization of strictures, monitorization of the therapeutic response, and diagnosis of postoperative recurrence (POR) [1,11,12,13,14].

Elastography is an ultrasound-based technique that can measure tissue stiffness [4,15,16]. It has been widely used for the assessment of different tissues, such as liver fibrosis and, in the last years, it has been explored as an imaging biomarker to measure fibrosis in patients with IBD [16,17,18]. However, the poor standardization of the technique, the existence of different elastography modalities (strain ratio or shear wave), and the fact that measures can be subject to the distance between bowel loops and ultrasonographic probes represent the main limitations for its generalization.

## 4. Intestinal Bowel Ultrasound in Patients with Crohn’s Disease

### 4.1. Intestinal Ultrasound for Detecting Inflammatory Lesions in the Bowel

According to the current ECCO-ESGAR and EFSUMB recommendations, IUS can be used as a complementary tool during the diagnosis of CD, contributing to determine its phenotype [1,19]. It can also be used as an alternative to endoscopy for detecting relapse or monitoring response to medical treatment during follow up [1,19]. There are no current studies showing the superiority of one cross-sectional imaging modality versus the other. Therefore, the recommendation of using IUS at diagnosis, versus computed tomography enterography (CTE) or magnetic resonance enterography (MRE), should be surrogated to the local expertise.

In the clinical scenario, a number of relevant features should be used, including: [4] bowel wall thickening, alteration of the bowel wall echo-pattern, loss of bowel stratification, increased vascularization, decreased bowel peristalsis, fibro-fatty proliferation, enlarged lymph nodes, and/or abdominal free fluid. Considering the transmural CD inflammation, CD complications such as presence of strictures, fistulae, or abscesses can be detected.

**Figure 2:**
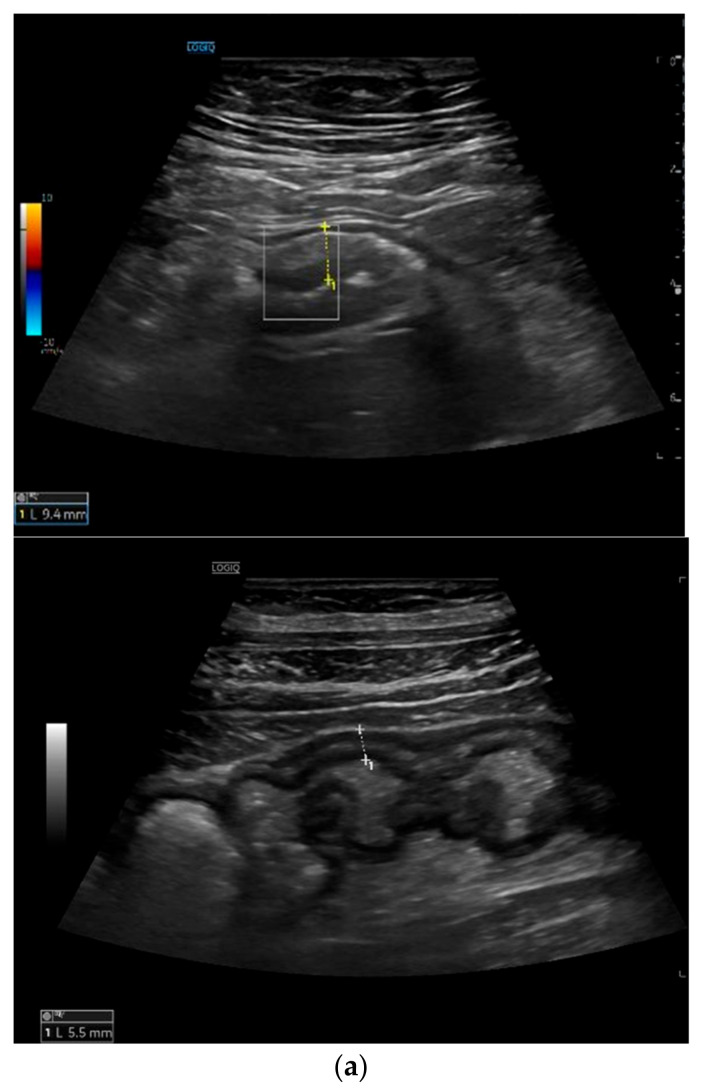

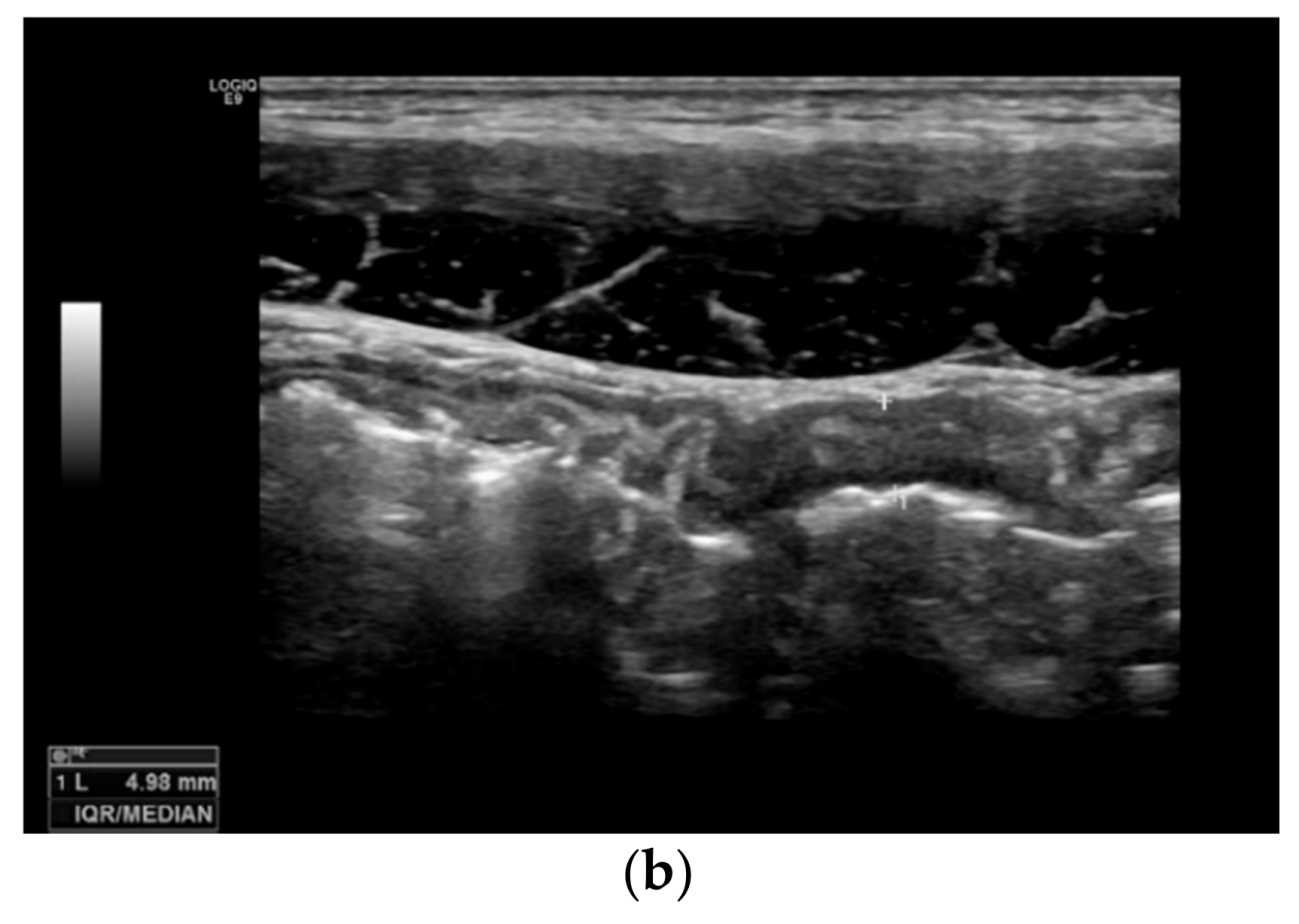
Measurement of bowel wall thickening and bowel wall stratification: Bowel wall thickening is the most important IUS parameter in IBD patients. The most commonly used cut-off value is 3 mm. The bowel wall stratification can be focally or extensively disrupted or lost due to inflammation. (**a**) Bowel wall thickening with preserved layer stratification. (**b**) Bowel wall thickening with loss of stratification.

Bowel wall thickening (BWT): this parameter measures the distance from the interface between the lumen and the mucosa layer and the interface between the serosa layer and the proper muscle. It should be measured in the anterior wall of the bowel (or where it is better visible) in the longitudinal direction, avoiding haustrations and mucosal folds (see Figure 2) [19]. Bowel wall thickening is the most important and most used IUS parameter for the diagnosis of CD [19]. A cut-off value of 3 mm has shown a sensitivity and specificity of 89% and 96%, respectively. A cut-off value of 4 mm or more showed a sensitivity of 87% and a specificity of 98% [20].

Bowel wall echo-pattern or bowel wall stratification (BWS): the bowel wall has a clearly defined multilayered aspect, but stratification can be focally or extensively disrupted or lost due to inflammation (see Figure 2). Disruption of stratification is associated to higher degrees of inflammation.

**Figure 3:**
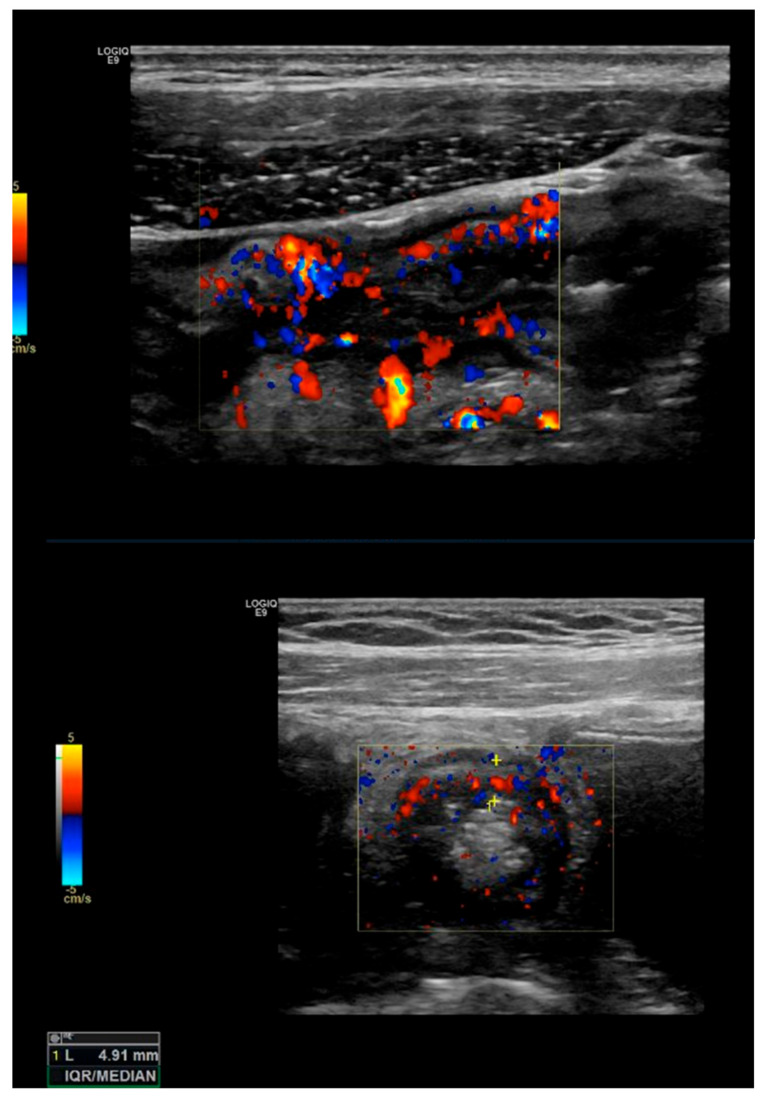
Bowel vascularization: Bowel wall vascularization can be measured by color or power Doppler US. The Limberg score is widely used for the assessment of mural and extramural flow. In the picture, an increased bowel vascularization (Limberg score grade 3) can be visualized in the inflamed bowel.

Bowel vascularization or bowel wall flow (BWF): vascularization can be measured at the thickened segments, by color or power Doppler US, using special presets of the machine optimized for slow flow detection [19]. The color Doppler flow is most widely measured by the Limberg score, which is a semi-quantitative score: [21] grade 0 represents a normal bowel wall with no thickening, well-delineated mural stratification, and no mural flow (no color Doppler signal); grade 1 is defined by a thickened bowel wall without vascular signals, grade 2 by the presence of short stretches of vascularity, grade 3 by the presence of longer stretches of vascularity, and grade 4 by vascular signals extending into the surrounding mesentery (see Figure 3).

Peristalsis: presence of peristalsis helps to differentiate the small bowel from the colon. IUS can evaluate peristalsis and compressibility of the bowel, the small bowel peristalsis being diminished in cases of wall stiffness. Lack of quantitative tools and inter-patients and inter-intestinal segment variability represents the main drawbacks of this item.

**Figure 4:**
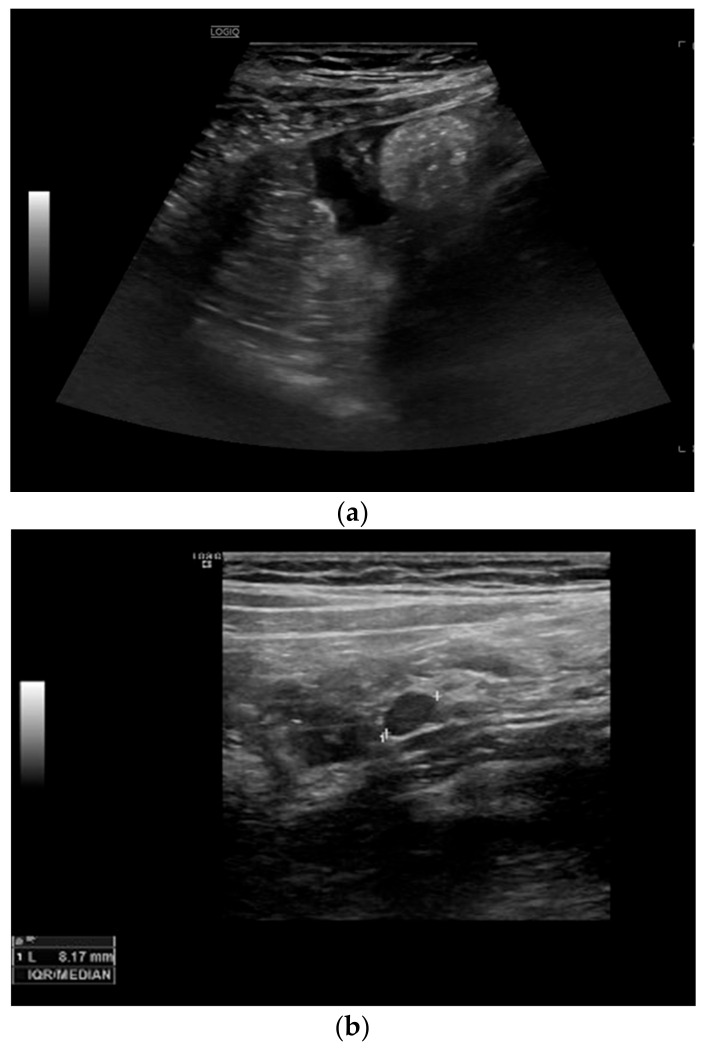
Extraintestinal ultrasonographic findings. (**a**) Presence of free fluid. (**b**) Detection of a lymph node and presence of fibro-fatty proliferation (echogenic peri-enteric fat).

Among the extraintestinal features of active CD, we can detect the presence of free fluid in the peritoneal cavity, enlarged mesenteric lymph nodes, and fibro-fatty proliferation (echogenic peri-enteric fat). Detection of free fluid close to the affected bowel segments is a non-specific but common finding. Enlarged mesenteric loco-regional lymph nodes are not specific of CD and can be present in other abdominal pathologies, but they are correlated with young age, early disease or disease with shorter duration, and with the presence of fistulae and abscesses [22,23]. Fibro-fatty proliferation or echogenic peri-enteric fat looks like hyperechoic tissue surrounding the diseased bowel and can be observed in patients with long-standing CD (see Figure 4).

**Figure 5:**
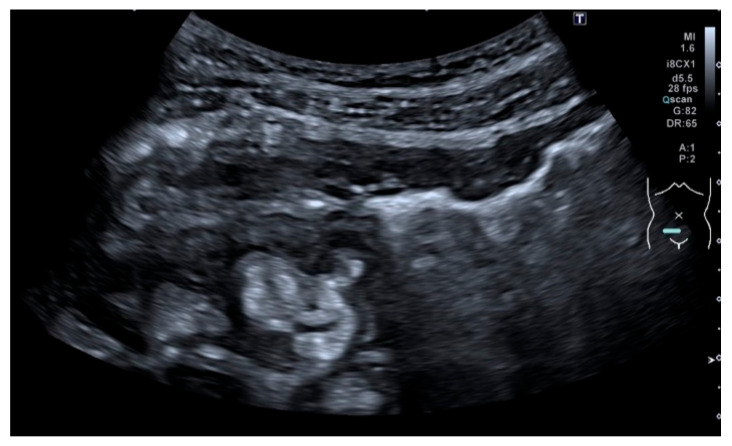
Detection of complications: stricture with pre-stenotic dilation: Strictures are characterized by a wall thickening with a narrowed lumen, with or without a dilatation of the proximal loop (pre-stenotic dilatation).

**Figure 6:**
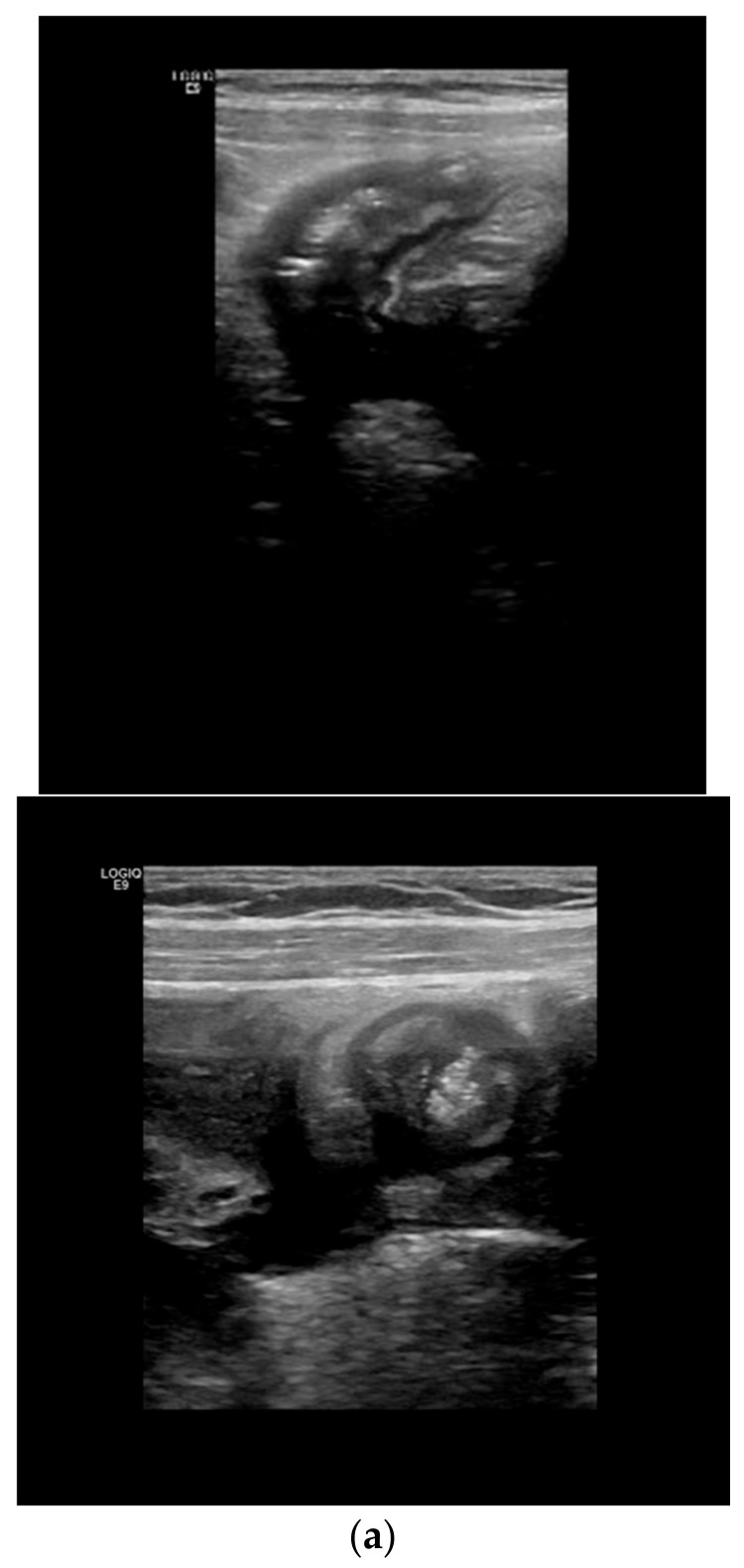

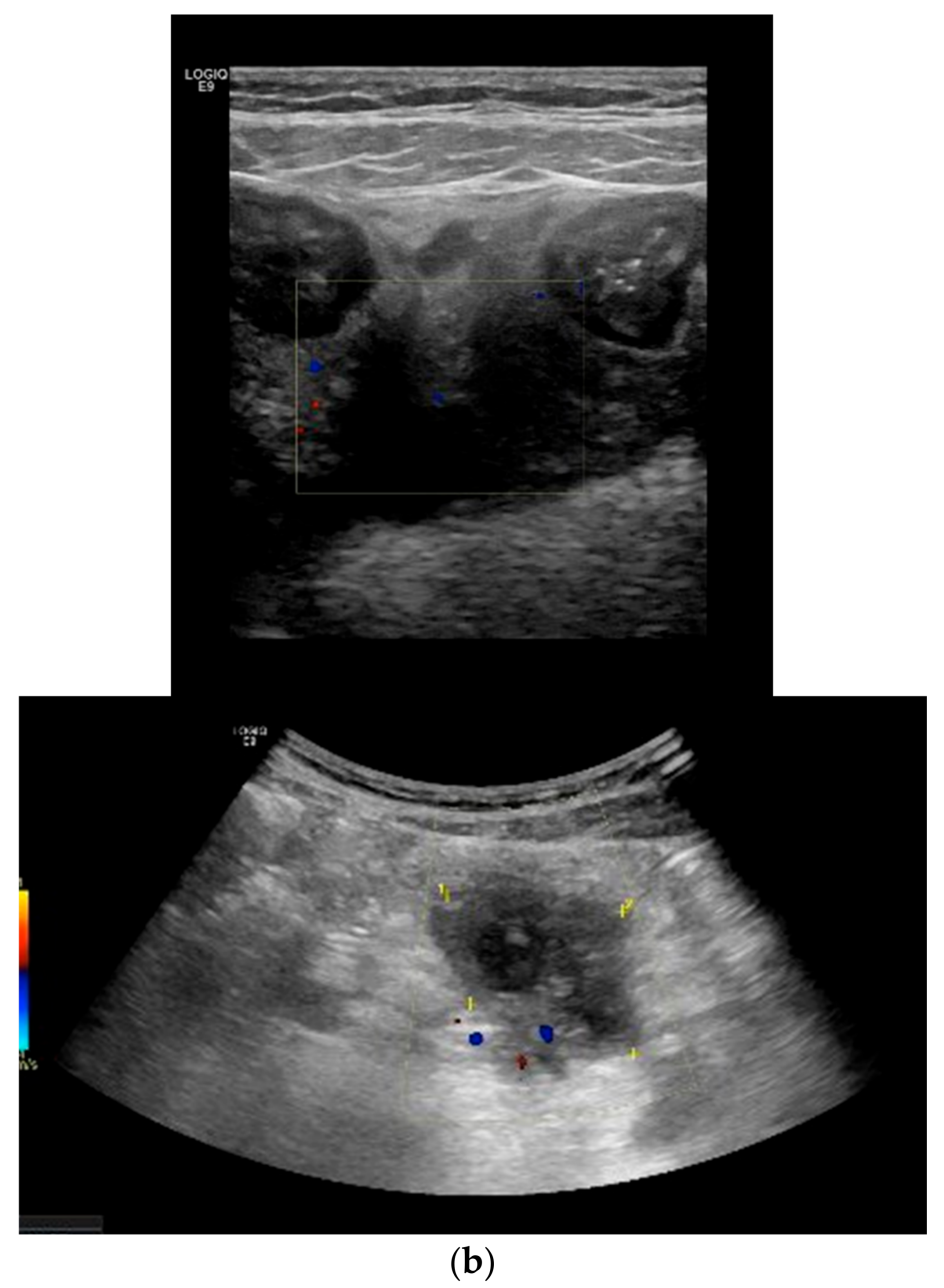
Detection of complications: fistulae and abscesses: (**a**) The fistulae are seen as hypoechoic peri-intestinal areas with a diameter <2 cm with or without internal gaseous artifacts. (**b**) Abdominal abscesses are seen as hypo-anechoic lesions containing fluid and gaseous artifacts, posterior enhancement, irregular margins sometimes within fibro-fatty proliferation, without vascular signals in color Doppler.

Depending of the CD phenotype, different ultrasonographic characteristics can be identified. The presence of ulcers can be visualized as depressions in the mucosal layer. Strictures are characterized by wall thickening with a narrowed lumen, with or without a dilatation of the proximal loop (pre-stenotic dilatation) (see Figure 5). The fistulae are seen as hypoechoic peri-intestinal areas with diameter < 2 cm with or without internal gaseous artifacts. Abdominal abscesses are seen as hypo-anechoic lesions containing fluid and gaseous artifacts, posterior enhancement, irregular margins sometimes within hypertrophic mesentery, without vascular signals at color Doppler (see Figure 6). An inflammatory mass is seen as an irregular hypoechogenic lesion, with vascular signals at color Doppler.

The METRIC trial, a British multicenter study, analyzed the accuracy of MRE and IUS in patients with a new diagnosis of CD or an established disease with a suspected relapse [24]. They found that both techniques were highly accurate for detecting small bowel CD. However, the sensibility and specificity of MRE was significantly higher than that of IUS (sensitivity for presence of CD with MRE 97% and with IUS 92%, p 0.027; sensitivity for small bowel disease extent with MRE 80% and with IUS 70%, p 0.025; specificity for small bowel disease presence with MRE of 96% and with IUS 84%, p 0.054; specificity for small bowel disease extent of MRE 95% and of IUS 81%, p 0.039). A prospective Italian study compared the diagnostic accuracy of IUS versus MRE in combination with colonoscopy for the assessment of disease activity and complications in 234 consecutive suspected CD patients [25]. IUS was less accurate than MRE in defining CD extent, whereas the concordance in terms of CD location between the two procedures was high. MRE also showed a fair concordance with IUS regarding strictures and abscesses, with better detection of entero-enteric fistulae.

### 4.2. Intestinal Ultrasound for Detecting Stricturing Crohn’s Disease

Different definitions of stricture have been published hampering an efficient communication in the IBD community. It limits the development of clear inclusion criteria and endpoints to measure treatment efficacy in clinical research. A recent international expert-based initiative proposed a standardized definition by ultrasound, including luminal narrowing (luminal diameter < 10 mm), wall thickness (>3 mm), and pre-stenotic dilation (lumen diameter > 25 mm) [26,27].

A recent systematic review analyzed the accuracy of the different cross-sectional imaging techniques for detection of strictures in patients with CD [27]. They only included studies with histopathological results, given that there is no validated gold standard for the assessment of inflammatory or fibrotic stricture. IUS sensitivity for stricture diagnosis was 80–100% with specificity of 63–75%. An increased accuracy was observed with the use of SICUS, with sensitivity rates of 88–98% and specificity rates of 88–100%. Similar sensitivity (75–100%) but a higher specificity (91–96%) was observed with MRE.

Strictures are characterized by different degrees of inflammation and fibrosis. The identification of these two components would improve the quality of IBD management: [1] medical treatment would be advised in patients with predominantly inflammatory strictures while endoscopic balloon dilation or surgery would be the best choice for patients with more fibrotic strictures. However, the available ultrasonographic techniques are not accurate enough to determine the degree of inflammation and fibrosis of a stricture. However, the presence of loss of stratification and hyperemia on color Doppler ultrasound or CEUS would be associated with the presence of inflammatory components at the stricture.

The use of CEUS would also be useful for the characterization of strictures in CD. A study in 25 patients with CD strictures using a dichotomized score (inflammatory versus fibrotic) found a good correlation with pathology for both inflammation and fibrosis [14]. Published data yield promising results with the use of elastography as a tool for the assessment of fibrosis, showing a good correlation between elastography and the degree of fibrosis [18]. A small study proposed a combination of elastography and assessment of bowel vascularization using the Limberg score for detecting intestinal fibrosis in patients with CD [28].

Waiting for further scientific evidence, the association of the basic IUS examination and Doppler with SICUS, CEUS, and/or elastography may increase the accuracy of IUS in detecting and characterizing CD strictures in clinical practice.

### 4.3. Intestinal Ultrasound for Detecting Penetrating Crohn’s Disease

The different cross-sectional imaging techniques have a high accuracy for the detection of penetrating CD such as fistulae or abscesses. A meta-analysis showed similar sensitivity of IUS, MRE, and computed tomography (CT) for the detection of fistulae [29]. IUS diagnostic accuracy for the detection of fistulae had a sensitivity of 67–87%, with specificity of 90–100%. IUS accuracy for detection of abscesses was slightly lower, with a sensitivity of 84–93% and specificity of 90–93% due to the more difficult detection of deep abscesses such as retro-gastric, deep pelvis, distal sigmoid, and rectal abscesses with IUS. In these locations, the accuracy of MRE was better.

The use of CEUS helps in the detection of penetrating complications, as it allows the differentiation between abscess and inflammatory masses and delimitates fluid cavities [1].

IUS is a useful and safe technique to guide drainage or aspiration of clinically relevant collection when there is no bowel or other anatomical structures interposed.

### 4.4. Intestinal Ultrasound for the Assessment of Response to Treatment in Crohn’s Disease

The role of IUS for the assessment of treatment response in CD has been widely studied. In a prospective study, patients receiving anti-TNF presented an improvement of IUS parameters (bowel wall thickness decreased in 46% of patients, reduction of color Doppler flow grade in 42% of patients, and disappearance of transmural complications in 50% of patients including a small mesenteric collection) that were significantly related to clinical and biological response [30]. Despite these positive findings, IUS parameters did not return to normal in a high proportion (71%) of patients with partial or complete clinical-biological response. A study after a year under anti-TNF showed that the ultrasonographic response after 12 weeks of therapy is more pronounced and predicts 1-year ultrasonographic response [31]. In the same way, a multicenter German prospective study, the TRUST study, included 234 patients with active CD and monitored treatment response [32]. After 3 and 12 months, IUS showed significant improvements in almost all parameters.

CD being a transmural disease, the assessment of transmural healing (normalization of IUS parameters) seems a more robust goal than mucosal healing in the treatment of patients with IBD. In a two-year observational longitudinal study, steroid-free clinical remission, mucosal healing, and transmural healing were assessed by coloscopy, IUS, and MRE [33]. Steroid-free clinical remission was achieved in 24/40 patients (60%), mucosal healing in 14/40 (35%), and transmural healing based on IUS and MRE in 10/40 (25%) and 9/40 (23%), respectively. There was a poor agreement between the steroid-free clinical remission and the presence of transmural healing (k = 0.27 for IUS and 0.29 for MRE; *p* < 0.01), and a good agreement between mucosal healing and transmural healing (k = 0.63 for IUS and k = 0.64 for MRE; *p* < 0.01). Transmural healing has been associated with better long-term clinical outcomes than mucosal healing, even after discontinuation of biologics [34].

The STARDUST trial, the first interventional multicenter sub-study using IUS in patients with CD, assessed IUS response to ustekinumab at weeks 4, 8, and 16 using a central reading system [35]. Four IUS variables were studied: bowel wall thickness, bowel wall stratification, vascularization, and fibro-fatty proliferation. IUS response to ustekinumab was detected as early as week 4, and a clinically meaningful percentage of patients achieved transmural healing, primarily in the colon, at week 16. Very recently, a multicenter Italian study assessed IUS to monitor CD activity in 188 patients receiving different biological therapies (adalimumab, infliximab, vedolizumab, and ustekinumab) [36]. IUS parameters of the study (bowel wall thickness, lesion length, bowel echo-pattern, vascularization, and transmural healing) were collected at baseline and after 3, 6, and 12 months of therapy.

IUS could be a valuable tool to detect early response to treatment in CD. The performance of SICUS in CD patients to assess response to anti-TNF showed that the presence of ultrasonographic complete response was associated with better long-term outcomes than partial or no response [37].

### 4.5. Intestinal Ultrasound for the Assessment of Postoperative Recurrence in Crohn’s Disease

The gold standard for the assessment of POR in patients with CD is ileocoloscopy [1]. Fecal calprotectin, IUS, MRE, and small bowel capsule endoscopy are considered non-invasive alternatives to detect POR [1]. Postoperative recurrence can be accurately detected using IUS by assessing bowel wall thickness at anastomosis one year after surgery (cutoff value of bowel wall thickness > 3 mm) [19]. The use of IUS for this indication is mainly based on the expertise of the center. Probably MRI is more advisable than IUS for providing an anatomical map of lesions in patients with CD candidates to resective surgery.

A systematic review with meta-analysis including 10 studies showed a high sensitivity and specificity of IUS for the diagnosis of POR [8]. The pooled sensitivity and specificity of IUS in detecting POR were 0.94 and 0.84, respectively, with a diagnostic accuracy of 90%. The increased bowel wall thickness was the most common parameter and it seemed to correlate well with the severity of endoscopic recurrence, bowel thickness ≥ 5.5 mm being the best cutoff value to predict severe POR (Rutgeerts score ≥ 3). SICUS seems to be superior to standard IUS examination for detecting POR, with higher sensitivity but lower specificity, and an overall accuracy of 92%, while the accuracy of CEUS needs to be determined with further investigation [8].

In addition to bowel wall thickness, increased vascularization measured by Doppler signal or CEUS may be additional tools to predict POR [38,39]. An Italian group identified bowel wall thickness (cutoff value of bowel wall thickness > 3 mm) and power color Doppler at anastomosis assessed by IUS one year after surgery as predictors of early recurrence after ileo-colonic resection. However, a preserved stratified pattern of the bowel wall at the pre-anastomotic ileum one year after surgery did not decrease the probability of POR in this subgroup of patients [40]. There is also evidence of good correlation of SICUS with endoscopic findings six months after ileocolonic resection [41].

### 4.6. Intestinal Ultrasound for the Examination of Pregnant Women

ECCO-ESGAR guidelines recommend IUS and MRE without intravenous gadolinium as the safest techniques to examine pregnant women in whom IBD is known or suspected, regardless of the trimester [1]. A multicenter observational study of women with IBD undergoing IUS during pregnancy assessed the bowel visualization in four colonic segments and in terminal ileum, showing IUS as a feasible and accurate method to monitor IBD during pregnancy [42]. Bowel wall thickness was compared with fecal calprotectin in pregnant women < 20 weeks, with an adequate visualization of colonic segments in 116/127 (91%) and of terminal ileum in 62/67 (93%) of IUS examinations. Beyond 20 weeks, IUS visualization of terminal ileum decreased to 30/51 (59%). There was a positive correlation between bowel wall thickness and fecal calprotectin. Accuracy of IUS in pregnant women showed a specificity of 83%, sensitivity of 74%, and negative predictive value of 90% compared to fecal calprotectin.

## 5. Intestinal Ultrasound in Ulcerative Colitis

Colonoscopy with biopsies is the recommended examination to establish a diagnosis of ulcerative colitis (UC) [1]. The role of IUS in UC is less explored than in CD [43]. IUS can assess disease activity and disease extension based on the identification of bowel wall thickness with or without color Doppler in patients with UC. The main relative limitation of this technique is that the rectum cannot be assessed in all patients. A recent relevant publication, the TRUST&UC study, demonstrated that IUS is also suitable for monitoring the disease course and for assessing short-term treatment response [44].

In patients with UC, where inflammation is limited to mucosa, ultrasonographic features are: [19] bowel wall thickening involving mucosa and submucosa (increased > 4.0 mm), alteration of bowel wall stratification (the stratification is usually preserved because UC is not transmural, but it may be disrupted in cases with severe UC activity), increased vascularization, and loss of haustra.

The TRUST&UC study is the largest multicenter study for the use of IUS in patients with UC [44]. In this study, bowel wall thickness was the best parameter to detect disease activity, being increased in 88.5% of patients at inclusion. A thickening of the submucosa was observed, probably due to submucosal oedema in active UC. The study showed a quick improvement in bowel wall thickness and vascularization two weeks after treatment intensification, followed by clinical improvement. Other IUS parameters such as bowel wall stratification, mesenteric fibrofatty proliferations, loss of haustration, and free fluid improved significantly at week 12.

Data regarding the use of CEUS in the evaluation of UC are still scarce, but it may be helpful to assess response to therapy [19].

The assessment of fecal calprotectin and IUS in general practice seems to be a reliable combination of non-invasive tools for monitoring of patients with UC.

## 6. Development of Intestinal Ultrasound Scores for the Assessment of IBD

The use of IUS scores may help predict the risk of surgery and quantify bowel damage [19]. Regardless of the potential advantages of the use of IUS in daily IBD clinical practice, there is a lack of validated scores to implement IUS as a standardized examination [45,46].

A recent expert consensus proposed an International Bowel Ultrasound Segment Activity Score (IBUS-SAS) for patients with CD in a three-phased process [47]. In the first step, four IUS key parameters (bowel wall thickness, bowel wall stratification, hyperemia of the wall using color Doppler, and fibro-fatty proliferation) were identified by international experts using Delphi methodology. Subsequently, the consensus was combined with a blinded agreement study. Bowel wall thickness was the most important predictor of disease activity in IUS, with almost perfect interrater agreement and correlation with overall assessment of disease activity. The other three parameter showed moderate to fair agreement and stronger association with overall assessment of disease activity.

The Simple Ultrasound Score for Crohn’s Disease (SUS-CD) was recently validated [48]. Seven IUS variables were initially included (bowel wall thickness, length, color Doppler, strictures, fistulae, bowel stratification, and fibro-fatty proliferation) but five of them were excluded during the validation process. The final SUS-CD included only two IUS variables, bowel wall thickness, and color Doppler, which correlated well with endoscopic disease activity.

The Lèmann score was developed to measure the cumulative structural bowel damage in 2011, taking into account damage location, extent, and severity [49]. It is based on the assessment of stricturing lesions, penetrating lesions, and antecedents of surgical resection, based on anamnesis and endoscopic or imaging diagnostic tools. An Italian study of 2017 evaluated prospectively the concordance between ultrasonography-based Lèmann score and MRE-based Lèmann score, observing a high concordance between the two imaging techniques [50].

Concerning patients with UC, published studies use different cutoff values of bowel wall thickness for identifying active UC (>3 mm and 4 mm) [43]. Several IUS scores for UC have been proposed but, to date, only the Milan Ultrasound Criteria (MUC) has been validated [43,51,52,53]. The study for the MUC validation evaluated five initial IUS parameters, proposing a definitive score that includes bowel wall thickness and vascularization. The score correlated significantly with the Mayo endoscopic subscore, an MUC score > 6.2 being the best cutoff value to discriminate active and non-active UC patients [53].

## 7. Future Perspectives of IUS

Intestinal ultrasound in CD has been largely expanded in Europe and in some areas of Canada and Australia. Efforts to standardize the technique and to implement a structured reporting are ongoing. They will consolidate IUS technique as part of the routine and improve the communication between specialties. Of note, ultrasound still has a limited role in the United States [54,55]. The reasons for these geographical differences should be further explored and may be explained by differences in health systems.

We also anticipate that the evidence supporting the use of IUS in patients with UC will increase during the coming years, given the high availability of the technique, together with the relative facility to explore the colon.

## 8. Conclusions

In conclusion, IUS has become a first line technique for the diagnosis and monitoring of patients with IBD. For its progressive implementation in the daily practice of IBD units, adequate IUS training for gastroenterologists and a generalized use of standardized scores is needed.

## Data Availability

Not applicable.
